# Treatise on the Diseases of the Kidneys, and on the Alterations of the Urinary Secretion

**Published:** 1841-10-01

**Authors:** 


TRAITÉ DES MALADIES DES REINS, &c. Par *P. Rayer.* Tomes 1, 2, 3. Paris : Bailliere.

TREATISE ON THE DISEASES OF THE KIDNEYS, AND ON THE ALTERATIONS OF THE URINARY SECRETION, &c. &c. With an Atlas of Illustrative Drawings, in Folio. By *P. Rayer*, Physician of La Charité Hospital, Consulting Physician to the King, and Member of the Royal Academy, &c.

IN the Numbers of this Review for April and July 1839, we submitted to our readers' attention some of the more interesting contents of the first volume of this important work, in connection with another instructive book, then recently published on the same subject, by Dr. *Willis*—his Treatise on the Diseases of the Urinary Organs. Since that period M. *Rayer* has brought out a second volume last year, and a third during the course of the present one.

The work, although it would seem to be not yet completed, is altogether so admirable a production that we are anxious that its value should be known to the English student without delay; and, for this purpose, we intend to devote a tolerably long article to its review at the present time, and may probably recur to our theme on some future occasion.

The *first* volume gives an ample account of the healthy and morbid anatomy of the kidneys; it then treats at great length of the urine, its composition, the best mode of examining it, the various changes it undergoes in health and sickness, the different kinds of sediment which it is apt to deposit, and the connection between the morbid alterations of this secretion and those of the blood and other fluids of the body: nearly 200 pages are devoted to the consideration of these topics. The rest of the volume is occupied with a description, first of wounds of the kidney, and secondly of common inflammation of the organ, or simple *nephritis;* the author illustrating at great length the relations which may be traced between this disease and the morbid states of other organs, as of the ureters, of the bladder, urethra and prostate gland, of the encephalon and of the spinal marrow, of the digestive apparatus, of the organs of circulation and respiration, of the skin, and of the lymphatic or absorbent system; and closing with a chapter on the connection of simple nephritis with febrile disorders.

The *second* volume commences with a description of those forms of nephritis that are induced by the absorption of morbid poisons and of purulent matter into the system, by certain of the exanthemata, by gout, and by rheumatism; but five-sixths of its pages are occupied with a most minute and elaborate account of that peculiar form of renal disease, to which the appellations of albuminuria, albuminous nephritis, granular degeneration of the kidneys, morbus Brightii, &c. have been given, and which of late years has excited so much attention in the medical world.

The *third* volume treats of *pyelitis,* or inflammation of the pelvis and calices of the kidney, and the relations between this not unfrequent disease and various other disorders, more especially those of the bladder and urethra, and those of the nervous system; then successively of *perine-*

*phritis*, or inflammation of the investing membranes of the kidney, of the different kinds of renal fistulæ, of renal hæmorrhages and apoplexy, of hypertrophy and atrophy of the kidneys, of *hydronephrosis* or dropsy of the kidney from obstruction to the escape of urine along the ureters, of the development of numerous morbid growths and alterations, such as cysts, cancer, melanosis, entozoa, &c.; and lastly, of various anomalies, congenital or acquired, as to the number, the relative position, the displacement, and so forth, of the kidneys.

By this summary of its contents, the reader will be able to judge for himself of the wide and extensive field of pathological research which a knowledge of the present work holds out to him; and, although here and there it may be capable of compression with advantage, we can confidently assure the studious physician that it will amply, aye more than amply, repay the labour of a most diligent perusal. In our opinion, it is perhaps the very ablest work on practical pathology, which has issued from the French press since the publication of *Laennec's* immortal work, with the exception of some of M. *Andral's* admirable writings. M. *Rayer* had already acquired an European reputation by his elaborate treatise on cutaneous diseases; the present one will greatly extend and advance it; and we most fervently trust that he will be long spared to gain fresh laurels in the peaceful pursuit of professional fame.

As it is utterly impossible to embrace within the limits of one article even a passing notice of the numerous subjects, each elaborately discussed, in the three volumes now before us, we purpose to select that one, perhaps the most interesting of all, which involves a consideration of that curious disease of the kidneys, in which the urine is invariably found to contain albumen, as well as to undergo other remarkable changes in its chemical and physical properties. This peculiar affection was first accurately described and discriminated by our own distinguished countryman, Dr. *Bright*, under the appellations of *diseased kidney in dropsy, and renal disease accompanied with secretion of albuminous urine;* M. *Rayer* prefers the term *albuminous nephritis;** and under this term we shall now proceed to give a condensed view of his elaborate description.

---

* Our author, alluding to the objections that have been made to the term *nephritis* being applied to a disease, which has not been proved to be essentially and invariably inflammatory in its nature, and which does not require, in numerous cases, an antiphlogistic treatment, states that in his opinion there is always more or less phlogistic action present during the early and acute stage of the malady, and that the physician is not more likely to be misled as to the remedies to be employed by using the term nephritis, than the surgeon is by that of cystitis in the treatment of various chronic diseases of the bladder. Then, as to the objection that the urine is *sometimes* albuminous in simple nephritis and other disorders of the kidneys (a fact which he has long admitted to be quite true), M. *Rayer* thinks that the very circumstance of this morbid phenomenon being only occasional in these cases, whereas it is constant in the disease now under consideration, is in itself an argument in favour of characterising it (the latter) by the appellation of *albuminous.* He very justly remarks that physicians have too long overlooked the acute form of the disease, which so often occurs after attacks of scarlatina, and not unfrequently from mere exposure to cold, as well as from

### ALBUMINOUS NEPHRITIS

Is chiefly characterised, during life, by the presence of a notable quantity of albumen, with or without sanguineous globules in the urine, by a diminution at the same time in the normal proportion of its salts and of the urea, by a diminished specific gravity of the fluid, and lastly by the co-existence or subsequent development of anasarca and various kinds of internal dropsy. The disease may be either acute or chronic, febrile or apyretic.

M. *Rayer* describes *six forms*—two of the acute and four of the chronic disease—characterised, the one from the other, by different pathological features; but it is to be remembered that the various morbid alterations may be found united in one case, when the disease has attacked, successively and at longer or shorter intervals, different portions of the two kidneys. He has never met with a case in which one kidney alone has suffered, although the two are often unequally affected.

*Anatomical characters of the first form.*—The size and weight of the kidneys are considerably increased—from 4 ounces, their ordinary weight, to 8 or even 12 ounces—their consistence is greater, but is not indurated; their surface presents a morbid red hue, and appears spotted over with a number of small red points, of a deeper colour than the rest of the organ. On making an incision into the kidney, we find that its increase of bulk is owing to a tumefaction of its cortical substance, which exhibits numerous red spots similar to those visible on the surface, and which, according to *Rayer's* researches, correspond with the glands of *Malpighi*, highly injected with blood. The tubular substance of the kidney, compressed between the tumefied prolongations of the cortical substance, is of a duller red, and its striæ are less apparent, than in the healthy condition. The mucous membrane of its pelvis and calices is sometimes injected, and exhibits vascular arborisations on its surface.

We rarely have an opportunity of observing this first stage of the disease, as it seldom proves fatal until a later period. We must be careful to distinguish it from mere hyperæmia of the kidney,—a not unfrequent concomitant of complaints of the heart—and also from simple nephritis: in this last disease the kidney is always harder and redder, and almost always exhibits some purulent points disseminated through its substance.

*Second form.*—The volume and size of the kidneys are still increased; their consistence is not quite so great, as in the first form. The lobules

---

the abuse of spirituous liquors, and which is so generally accompanied with what has been long called *inflammatory* or *plethoric dropsy.*

After stating his own objections to the appellations of *granular degeneration of the kidneys* proposed by Dr. *Christison*, and of *albuminuria* by M. *Martin Solon* and Dr. *Willis*, M. Rayer says, "I should have been much disposed to adopt the denomination of *maladie de Bright*, as consecrating the discovery of this celebrated physician, if it had not seemed to me preferable to give the disease a significative pathological name."

are often more distinct than in health. But the special character of this form is the very remarkable commixture of anæmia and hyperæmia, which gives rise to a marbled appearance of the surface of the organs, produced by red patches disseminated on a yellowish white ground. On cutting the kidney, its cortical substance is observed to be still swollen, but it is now of a pale yellowish hue spotted with red, and there is a well marked line of demarcation between it and the tubular substance, the colour of which is of a reddish brown *assez vif*.

*Third form.*—The affected kidney is still larger and heavier than in health, but it does not present any red patches or marbled appearance: its cortical substance, both on the surface and when cut into, exhibits a tolerably uniform pale or whitish-red colour, verging to yellow; in some cases the hue is more pale still, and very nearly resembles that of the flesh of the eel. Here and there, we observe minute vessels injected with blood, and more rarely small brownish patches, or large white granulations, produced by an old deposition of plastic lymph. The papillæ of the tubular substance of the kidney often exhibit red indurations; and the mucous membrane of its pelvis and calices is occasionally thickened, and here and there injected. It is to be remembered, however, that the lesions now described cannot be considered as characteristic, seeing that they are to be also noticed in some cases of simple, not albuminous, nephritis.

*Fourth form.*—This has been designated by Dr. *Bright* the *granulated texture of the kidneys*. The increased volume of the organ continues; its surface, usually of a yellowish colour, is dotted, and sometimes covered with minute spots of a milky white with a yellowish hue, which are often elongated, and give very much the appearance as if small portions of milk curd had been dropped irregularly on the surface of the kidney. These granulations are usually most numerous and distinct at the two ends of the organ; they are not prominent, for the surface of the kidney is quite smooth to the touch: they are imbedded in its cortical substance. If we cut the kidney from its convex to its concave side, its cortical substance exhibits, as in the second and third forms of the disease, a pale yellow colour which contrasts strongly with the red hue of the tubular portion; it (the former) is swollen and occupies a considerably larger space than in health, especially in its prolongations between the cones of the latter. The milky white spots, or granulations of Dr. *Bright*, instead of appearing rounded and distinct from each other, as they usually do, on the outer surface of the organ, now look like irregular flocculent lines, which seem to be continuous with the divergent striæ of the tubular cones. When the incision has been well made in the direction of these striæ, this arrangement is very distinctly seen, especially at the periphery of the kidney and the base of the cones, where the granular degeneration is usually most conspicuous.

In some cases the granulations, although very distinct on the surface of the kidney, can scarcely be observed in the substance of the cortical structure; while in other cases they are scattered through every portion of it, even to the small prolongations which penetrate into the bases of the tubular cones.

The granulations become distinct, if the kidney has been macerated for some time in water; their dull white colour stands out then more obviously from the surrounding cortical substance. M. *Rayer* alludes to two cases of albuminous dropsy in children, where they were unusually large and of a rounded shape.

*Fifth form.*—The kidney is larger, heavier, and has its lobules more distinctly marked than in health. I cannot, says our author, give a better idea of the peculiar aspect which it exhibits than by saying that it seems as if a great number of the minute grains of semolina? (*semoule*) were sprinkled on its surface under the investing cellular membrane.

These minute grains, very distinct from the yellow particles sometimes observed in the cortical substance, are the small granulations of plastic lymph which we meet with accidentally in this and in other forms of nephritis.

*Sixth form.*—This corresponds with the third variety described by Dr. *Bright*. The diseased organ is sometimes larger, but often smaller, than in health; it is hard and more or less irregular or tuberculated. We observe few, or perhaps none at all, of the milky spots or granulations on the surface of the affected kidney; but a certain number is always found, when an incision is made into the cortical substance. The surface of the kidney is indurated, corrugated, and mamillated; but, although sprinkled over, perhaps, with minute asperities, it does not exhibit the genuine granulations of Bright. In some cases, it must be confessed that the anatomical characters of this form of the disease are so closely alike to those observed after simple chronic nephritis, that it would be scarcely possible to point out the distinction between them, if we did not take into account the phenomena present during the life of the patient. In this advanced stage of the disease, the investing membranes of the kidneys are almost always thickened, at least in several points, and strongly adherent.

### *Symptoms of Albuminous Nephritis.*

*The Acute form.*—This is of not unfrequent occurrence in children after an attack of scarlatina, especially during certain epidemics; it is also occasionally observed in adults after exposure to sudden changes of temperature, from heat to cold and damp weather. It often commences with shivering, followed by the usual symptoms of pyrexia; the urine is observed to be scanty, and is of a deep brown or reddish colour, not unlike to the washings of fresh meat. It is always acid; its specific gravity is often above, and seldom below, that of healthy urine, the proportion of the urea and of the salts being generally not much, if at all, affected. When allowed to rest, it deposits filamentous flocculi, which seem to be formed by the fibrinous part of the blood; for a certain quantity of this fluid is usually blended with the urine.

The odour of the urine is feebly urinous; at the end of twenty-four hours, it often exhales a peculiar smell, not unlike to that of beef-soup. If we examine with the microscope this albuminous and sanguinolent urine, when first voided, we observe that it holds in suspension a great number of blood-globules, occasionally also globules of mucus, and always some minute lamellæ of epithelium. Left to rest for some time, this urine exhibits a sediment composed almost entirely of these globules and lamellæ, and sometimes also of fibrinous filaments: a deposit of uric acid is rare; it is more frequent in the chronic form of the disease. We have already said that the specific gravity of the urine in the acute form of albuminous nephritis is usually not much affected; if pleuritis, pneumonia or other inflammatory disease be present at the same time, the tendency will be to an increase, rather than to a diminution, of the specific gravity. Occasionally, but certainly not in most cases, as alleged by Dr. *Christison*, there is a certain degree of distress, and even of pain in voiding the urine; this is generally owing to a sympathetic irritation of the bladder, but, in a few rare instances, to the difficult passage of fibrinous concretions along the urethra. There is almost always a dull aching and sense of uneasy constriction or weight in the loins; these symptoms are sometimes greater on one side than on the other; but they are never so severe as in simple nephritis. In no case of albuminous nephritis has M. *Rayer* ever observed any retraction of the testicles, or heard the patient complain of pains darting in the direction of the ureters.* On the whole, therefore, the local symptoms of the disease must be considered as inconstant and unsatisfactory.

Scarcely has the alteration in the urine fairly developed itself, before symptoms of dropsical effusion make their appearance in some cases with extraordinary rapidity; in short, this is one of the most constant and most characteristic signs of the disease. It usually is first noticed as a puffiness of the eyelids or of the whole face; at other times it primarily affects the limbs, and extends upwards to other parts of the body; occasionally it is erratic, ceasing in one part and making its appearance in another part. The skin is usually hot, and does not pit readily except on strong pressure.

There is always more or less pyrexia of the whole system present; the tongue is furred, the stomach sometimes rejects every thing, and occasionally also the patient is troubled with a cough.

If blood be drawn at this time it is very generally found to be buffy, and sometimes remarkably so; the serum is occasionally milky; but this appearance is much more frequent in the chronic than in the acute form of the disease.

At the commencement of the disease the serum coagulated by heat or by the addition of an acid has its natural consistence; a few days afterwards, the coagulum obtained in this way is found to be less firm than before. The specific gravity of the serum is generally observed to be inversely as the urine is charged with albumen; the greater the quantity of this element in the urine, the lower is the specific gravity of the serum of the blood. M. *Rayer* has several times noticed that, a few days after blood has been drawn, the specific gravity of the serum has increased, when the urine had become less albuminous in consequence of the reme-

* Dr. *Christison*, however, states that he has observed this symptom—pain extending down the inside of the thighs and to the genital organs—in several cases of albuminous nephritis.

dies employed. He has not succeeded in detecting urea in the blood at this early stage of the disease; but he admits that it may be so, and appeals to the observations of *Christison*.

The cure of acute albuminous nephritis is sometimes very rapidly effected, especially when the disease comes on after scarlatina, or during the latter months of pregnancy. It is usually announced by a profuse perspiration, by a copious discharge of urine, by a sensible and progressive diminution in the quantity of albumen, and an increase in that of the urea and the salts contained in it, by the cessation of the feverishness, and the subsidence of the œdema of the face and other parts. If any dropsical effusion has taken place into the cavity of the thorax or of the abdomen, the cure will necessarily be protracted, even under the most favourable circumstances.

In a few cases, acute albuminous nephritis and the general dropsy, which is one of its principal phenomena, terminate fatally; death is usually announced by the rapid development of cerebral symptoms, or of some thoracic inflammation.

In other cases, the disease assumes a chronic character: the feverishness and the anasarca may quite disappear, and the patient seem to have recovered his health; but the urine still retains it albuminous composition. As long as such is the case, the physician cannot consider the cure as complete.

*Chronic Albuminous Nephritis.*—This is incomparably more frequent than the acute form of the disease: often it is the sequence or result of the latter; but, in the majority of instances, it has the passive character from its commencement.

In persons of a scrofulous constitution, or whose health has been damaged by former illness, I have observed, says M. *Rayer*, the urine—which a short time before exhibited no abnormal phenomena—assume all the characters observed in cases of chronic albuminous nephritis with general dropsy. This change sometimes comes on without any appreciable cause; at other times it seems to be attributable to the influence of cold and damp.

The urine, when first voided, is almost always slightly acid; but in a few cases it is neutral or even alkaline. It is always pale, and not unfrequently somewhat opaque, or like whey in which small whitish flocculi are suspended. The odour of the urine is faint, and very different from that of the secretion in health; its specific gravity is usually below the normal standard, and in some cases very considerably so. Examined with the microscope, this urine almost always exhibits numerous small thin lamellæ of a whitish colour, with which are often blended a mucous matter that is either amorphous or in the form of globules.

The want of transparency of the urine is sometimes owing to the presence of a fatty matter held in suspension; this may be removed by means of sulphuric æther; and then the urine becomes quite clear.

If we examine a phial filled with albuminous urine, we usually notice on its surface, and against the sides of the glass, a number of bubbles; and if we blow air with a tube into it, we immediately observe a multitude of large bubbles, like those formed in the same way in soapy water. The action of heat and of nitric acid at once produces the formation of an albuminous coagulum; this, in some cases, is so abundant as almost to solidify the whole mass. The yellow cyanuret of potassium and iron also coagulates albuminous urine, if it has been previously acidulated with acetic acid,

When this state of the urinary secretion exists, we very generally find that there is a certain degree of increased irritability of the bladder; for the patient has more frequent calls to pass water than in health, even when the actual quantity voided in twenty-four hours is below the normal standard; there is however seldom any pain felt in the loins, except perhaps when very firm pressure is made.

The alterations of the urinary secretion now described may continue for several months, before any symptoms of dropsical effusion into the cellular tissue or elsewhere make their appearance, and without being accompanied with any signs of constitutional disturbance, except debility of the muscular system and impairment perhaps of the appetite. But if the patients are not carried off by some casual illness, or by one of the secondary affections which so frequently supervene during the course of albuminous nephritis, we may with certainty predict that they will become sooner or later dropsical. The most frequent form of dropsy is unquestionably anasarca. In the morning the eyelids and face are observed to be puffy; and towards evening, if the patient has been standing much during the day, the feet and ancles also are generally swollen. The œdema of the lower extremities, connected with disease of the kidneys, does not, it would seem, so readily subside by rest in the horizontal position as when the dropsical effusion is dependent on some organic affection of the heart: this diagnostic difference should therefore be attended to.

M. *Rayer* says that, in several cases, he has observed that the nephritic anasarca was very sensibly and rapidly aggravated by exposure to cold air—more so than is ordinarily the case in other forms of the disease. Ascites is another form of dropsy that is not uncommon, more especially in those cases where there is co-existent disease of the heart or liver; hydrothorax and hydro-pericardium also are not unfrequent in the latter stages of the malady.

It deserves to be noticed that chemical analysis has occasionally detected in the serosity of these effusions a certain quantity of urea, independently of the albumen and saline matters usually discoverable in them.*

When albuminous nephritis has existed for a length of time, the *blood* is found to undergo remarkable changes. The proportion of the coagulum becomes much diminished, and that of the watery portion correspondently increased. On examining the serum by itself, we find that it contains less albumen and saline matter than in health, and that its specific

---

* *Nysten*, so far back as 1810, alludes to the presence of urea in the water of ascites. In 1833, M. *Guibourt* detected it in the case of one of M. *Rayer's* patients; and in 1834, Dr. *Barlow* detected it in the serum found in the ventricles of the brain in a patient whose urine was coagulable, and in whom the kidneys exhibited on dissection the granular degeneration, although no form of outward dropsy had occurred during life.

gravity is lower.* The researches of *Bostock*, *Christison* and *Gregory*, are very valuable on this subject. Occasionally it has a milky appearance, and looks very like whey.

If we examine with the microscope the blood of hydropic patients, who have been long affected with albuminous nephritis, the *red globules* are observed to be less numerous than in health; and mixed with these we find other globules of a white colour and of a larger size than the former. There can be no doubt that a certain portion of *urea* may be occasionally found in the blood; it is small indeed and very variable in quantity; but that it does sometimes exist cannot be disputed. The less the proportion that is contained in the urine, the larger will be the quantity discoverable in the blood. Whoever desires the most exact information on this and other points connected with the chemistry of the disease, must apply to Dr. *Christison's* work.

As to the proportion of the *fibrine*, the experiments hitherto made are insufficient to enable us to speak positively. The blood is often found to be covered with a buffy coat, with retracted and puckered edges as in acute rheumatism, even in the chronic form of albuminous nephritis; but M. *Rayer* is not inclined to agree with Dr. *Christison* that the quantity of the fibrine can be estimated from the thickness of this *couenne*. It is to be remembered that the size of the coagulum is usually considerably smaller than in health; and also that the buffiness of the surface is not unfrequently owing to the co-existence of some secondary inflammation of the abdominal or thoracic viscera.

Independently of the more striking phenomena of chronic albuminous nephritis which we have described, there are some others that deserve to be noticed—for example, the marked diminution of the perspiration, the dyspnœa, sometimes accompanied with vomiting, and often with a diarrhœa, which, however profuse it may be, never causes any sensible diminution of the dropsical effusion. The breathing is almost always embarrassed, either from existing bronchitis, or pulmonary œdema, or hydrothorax, or from some other morbid affection of the lungs or heart, either antecedent or consecutive to the disease of the kidneys. In a few cases cerebral symptoms come on; the rapid invasion of these is almost always the sign of approaching death.

The *duration* of albuminous nephritis varies much, from a few months to several years. It is always difficult to determine with exactitude the epoch of its commencement, especially in hospital practice, as it is usually for the consecutive dropsy that the patients apply for relief, and they are not at all aware that they have any other complaint.

Once that the characteristic alteration of the urinary secretion is ascertained to exist, the physician, as we have already stated, may with tolerable confidence predict that, unless the patient is carried off by some accidental malady, some form of dropsy or another will almost inevitably ensue. But when this will take place, and, after it does, how long the

* The medium density in health is 1028 or 9; during the course of the disease it falls to 1020, and sometimes, even as low as 1013; the diminution seeming to be directly proportionate to the quantity of the albumen in the urine.

patient is likely to survive, it is not possible to form any reasonable conjecture.

"In the greater number of cases," says M. *Rayer*, "the dropsy continues until death—presenting, like the disease of the kidneys, remissions and exacerbations at longer or shorter intervals of time, or perhaps occasional amendments so considerable and so durable that the patient is enabled to attend to his business without much interruption for several years, and until the disease, assuming a more active form, forces him to keep bed and then terminates fatally more or less rapidly, in consequence of the supervention of a secondary complaint, such as some cerebral affection, or pericarditis, or pleurisy, or pneumonia, or gangrenous erysipelas, or perhaps obstinate diarrhœa, with or without vomiting and general fever."

Much will depend, as a matter of course, or the general constitution of the patient's body, whether this be cachectic or not, and on various other considerations which will necessarily be taken into account in forming our prognosis of every serious malady. This leads us by a natural step to consider

*The Causes of Albuminous Nephritis.*

The exposure of the body to cold and damp, or whatever has the effect of suddenly checking perspiration, as for example drinking a cold fluid while the skin is hot or sweating, is unquestionably the most frequent cause of the *acute* form of the disease; hence it is most commonly observed in those who are subjected to rapid changes of the temperature, such as bakers, workmen in glass-works and distilleries, and convicts who are kept at forced labour out of doors. The action of cold and moisture is most remarkable, where the disease follows scarlet fever during the period of desquamation. It is to the same agency that we can generally trace the exacerbations and relapses of the disease, when it has been once checked by a judicious system of treatment.

The *chronic* form of the disease may be induced by a variety of causes; but the most frequent of these, at least in France, is habitual or long-continued exposure to cold and damp. The most of the cases treated by M. *Rayer* have occurred either in workpeople, whose occupation exposes them to wet, or in persons who have been living in unhealthy, cold, and damp dwellings. The intemperate use of spirituous liquors increases much the predisposition to albuminous nephritis. Dr. *Christison* is of opinion that three-fourths, or even a larger proportion, of the cases in Great Britain, are attributable to this most pernicious practice; but, as the vice of intemperance is not nearly so prevalent on the Continent, our author cannot judge of the accuracy of this statement. The frequency of the disease in hackney-coachmen, porters at wharfs, &c., may be no doubt explained in this way.

Again, whatever tends to render the system cachectic is sufficient to induce disease of the kidneys in certain cases. M. *Rayer* states that he has seen several instances in which pregnancy also appeared to have a real influence in the development of albuminuria; but he does not endeavour to account for it. Scrofulous and syphilitic disorders have unquestionably, according to his experience, powerfully predisposing effects, especially in those who lead an intemperate life, and are exposed much to the vicissitudes of weather at the same time; but he does not consider that gout, scurvy, or purpura, although the urine in these diseases is sometimes bloody and coagulable, have any decided tendency to produce a lesion of the kidneys. Pulmonary phthisis however seems to have a marked effect; so also have organic diseases of the heart, and likewise of the liver; although it must be confessed that the hepatic disease seems not unfrequently to be of quite as long standing as that of the kidneys—both being so often attributable to the same cause, the abuse of spirituous drinks.

M. *Rayer* is not satisfied, from the results of his own experience, that mercury can be regarded as an exciting cause of disease of the kidneys. He has for many years past been in the habit of using various preparations of this metal in cutaneous and hepatic affections, without having ever observed that it seemed to lay the foundation for any dropsical affection. That however mercury occasionally induces an albuminous state of the urine appears to be proved by the observations of Dr. *Wells* and Dr. *Blackall*. *Christison* also cites a case, which, in his opinion, goes to shew that the mercurial action has some influence on the development of the granular degeneration of the kidneys.

With respect to the sex and age, in which the disease is most frequent, it appears that it is unquestionably more common in males than in females, and its attacks seem generally to take place between the 20th and 40th year of life: it is of rare occurrence in old age.

*Diagnosis.*—The acute form of albuminous nephritis may, in most cases, be recognised without much difficulty, by the co-existence of an albuminous and often sanguinolent state of the urine with the rapid development of anasarca, and occasionally of dropsical effusion into some internal cavity. In no other acute disease do we meet with the re-union of these characters.

In a few cases no dropsical effusion takes place; but even then the peculiar state of the urine, accompanied with more or less febrile irritation and derangement of the general health, will be sufficient to diagnosticate the disease. Hæmaturia is perhaps the only complaint with which it may be mistaken; but in this we can generally recognise, at least with the microscope, the pure blood mixed with the urine; and, if such be not the case, then we observe fibrinous concretions or filaments floating in the water; the passage too of the urine is generally more or less painful, and the pain on one or on both sides of the loins is more severe than it ever is in cases of albuminous nephritis. It deserves to be noticed here, that in some cases—they are rare—of dropsy supervening upon scarlet fever, the urine will be found to contain neither any blood-globules nor albumen; but in by far the greater number of cases it is otherwise.

The *diagnosis* of the *chronic* disease is attended with much greater difficulty and incertitude than that of the *acute* form; whether we compare those cases that are accompanied with dropsy with cases where dropsy is produced from other causes, or, on the other hand, compare those cases of albuminous nephritis, in which dropsical effusion is not present, with certain diseases of the kidneys, or of other viscera, in which the urine either habitually or accidently contains a more or less considerable quantity of albumen.

When, in a patient who experiences only trifling if indeed any pain in the loins, the urine is found to be of a low specific gravity, and contains albumen with only a small proportion of urea and the urates, the presence of chronic albuminous nephritis may be regarded as certain, especially if there be no disease of the heart existing at the same time. Even when there is, the chances of mistake are but small indeed, and for the following reasons. When the kidneys experience a passive hyperæmia and congestion of blood, in consequence of the obstruction to the free circulation through them, occasioned by some existing disease of the heart or lungs, the urine is apt indeed to contain a certain portion of albumen and sometimes also of red globules; but the quantity of these foreign ingredients will be found to be very irregular at different times, and to be much diminished by quietude and repose in bed—a change which we do not observe to take place in genuine disease of the kidney. Moreover the specific gravity of the urine and the proportion of the urea and urates are not affected; at least to the same degree. The dropsical effusion too caused by disease of the heart usually commences in the lower extremities and extends upwards, whereas that arising from a lesion of the kidneys is often first perceived in the face.

In diabetes, the urine is sometimes found to contain albumen; but then, at the same time, saccharine matter exists in it.

Again, when the urine becomes charged with mucus or pus, in consequence of disease of the pelvis of the kidney, or of the bladder, the action of heat or of nitric acid upon it will discover the presence of albumen. It might be deemed unnecessary to mention this, were it not that cases of *pyelitis* (inflammation of the pelvis and calices of the kidneys) and of cystitis have actually been published as examples of albuminous nephritis—the authors forgetting that the mere presence of albumen in the urine is not an exclusive phenomenon of the latter disease, and overlooking at the same time the presence of mucus or of pus, which are seldom or never found in the urine of patients affected with genuine albuminous nephritis, as well as other characters which distinguish the former maladies.

"When, after a few days' indisposition," says M. *Rayer*, "a patient is affected with serious cerebral symptoms, or with repeated attacks of vomiting, without dropsy; if, at the same time, the urine is strongly charged with albumen, and is of a low specific gravity, and if we cannot detect any existing disease of the heart or of the pelvis of the kidney, or of the bladder or urethra, the existence of chronic albuminous nephritis may be regarded as more probable than that of a primary cerebral affection. And if it is ascertained that the patient has been exposed to wet and cold, or that he has been addicted to the abuse of spirituous drinks, or that he had been affected with dropsy some months before, the diagnosis of renal disease may be asserted with still greater confidence.

If, while the urine exhibits the characters already indicated, there is no existing disease of the heart or large blood-vessels, nor any affection of the liver, nor the state of pregnancy be present, and if, under such circumstance, anasarca or other form of dropsy comes on, we have strong reason to regard the kidneys as the source of the evil."

He confesses however that, in some complicated cases of disease of the heart and liver, it is exceedingly difficult, if possible, to determine whether the dropsical effusion arises primarily from the lesion of these organs, or from that of the kidneys.

The dropsies, which not unfrequently supervene on protracted intermittent fevers, especially on those of the quartan type, are of two sorts;—one depending upon disease of the kidneys, and characterised by an albuminous state of the urine; while the other sort, whose mode of production is not well known, seem to be more immediately connected with the aguish state of the system, being usually curable by means of bark and other febrifuge remedies.

In conclusion, we must here remind our readers that it is only by studying attentively the *ensemble* of a case, and not by dwelling upon any single and unassociated symptom, that we can hope to arrive at any accurate conclusions in our diagnosis of renal disease; "there is perhaps not one symptom of albuminous nephritis, either acute or chronic, that is not occasionally met with in other maladies, and yet it is equally true that there is a series of symptoms which belongs only to this disease, and by which its existence may generally be detected."*

*Prognosis.*—It is unnecessary to amplify much on this topic, as the scope of our remarks may be easily anticipated from many of the preceding observations. When the disease is acute, death sometimes very suddenly supervenes, in consequence of disease rapidly developing itself in the brain, or lungs, or pericardium. Hence the attention of the judicious physician will always be drawn to the state of these organs, as long as the urine remains albuminous or sanguinolent.

As to the chronic disease, it must always be regarded as one of very serious import; as long as the urine remains coagulable and of diminished specific gravity, the health of the person cannot be considered otherwise than very unsatisfactory, from the tendency that exists under such circumstances to development of various maladies, such as dropsy, pleuritis, cerebral affections, &c.

Any marked diminution in the quantity of the urine, when it is abnormal in composition, should always be viewed with especial suspicion, as this is not unfrequently the precursor of cerebral or thoracic mischief being established. The co-existence of disease of other organs, or a cachectic state of constitution, will necessarily render our prognosis in cases of albuminous nephritis more unfavourable: we shall recur to this topic when we treat of the complications of the malady, and shall therefore now proceed to that more interesting subject,

*The Treatment.*—The two chief causes which tend to develop albumi-

---

* M. *Rayer* mentions the thesis of M. *Tissot* in 1833, and that of M. *Desir* in 1835, as especially valuable in pointing out the various diseases in which the urine has been found albuminous. The milky state of the blood has been observed by *Trail* and *Hewson* in cases of simple nephritis, of hepatitis, of rheumatism and of asthma, as well as of granular degeneration of the kidneys: vide *Christison*, Ed. Med. and Surg. Journal, vol. xxxiii. Urea has been detected in the blood after attacks of simple nephritis, where the urine was not coagulable, and also in cases of atrophy of the kidneys.

nous nephritis, and the dropsy which is the consequence of it, being the sudden or prolonged impression of cold and moisture, more especially during the recovery from scarlet fever, and the abuse of spirituous liquors, the aim of the physician must be to guard against and counteract the influence of these agencies. And, although in the majority of cases professional assistance is not resorted to until the disease has existed for a considerable time, still there are some in which the practitioner, if he be upon his guard, may succeed in entirely preventing the establishment of the mischief. Hence the importance of examining occasionally the state of the urinary secretion during the course and stage of convalescence from attacks of scarlet fever, and of using all judicious means to preserve the patient from exposure to cold.

Among the poorer classes this precaution is the more necessary, for very obvious reasons.

With respect to the influence of intemperate habits, it has only been within the last few years that medical men have been aware of their tendency to induce a serious lesion of the kidneys. The liver and stomach have hitherto been considered as the organs that suffer most from intemperance; but it now appears that the kidneys are as liable to become diseased as they are. Whenever the urine of a person addicted to this vile custom is found to contain albumen, or when any œdematous puffiness is observed in the face, there is strong reason to suspect the existence of renal disease, especially when there is no existing affection of the heart or great bloodvessels. It is almost unnecessary to add, that if the habit is not abandoned by the patient, or if there be symptoms of co-existent phthisis or heart-disease, or when there is a venereal cachexia of the constitution, there is little or no chance of ultimate success for any mode of treatment.

Among the most useful of all remedies we must rank the detraction of blood, either from the arm, or by means of cupping over the loins. The quantity to be drawn will depend upon the severity of the febrile excitement, and the rapidity with which the dropsical affusion takes place. The frequency of the operation will be determined by the state of the blood drawn, according to the amount of its fibrinous portion and the thickness of the buffy coat. M. *Rayer* says that, as a general rule, it is safer to draw rather too much than too little during the acute stage of the disease.

If the pyrexia be not severe, one general bloodletting may be sufficient; but it is always well to follow it up with cupping over the renal region. Sometimes a troublesome erysipelatous inflammation appears round the wound in the arm or the cuts of the scarificator: but, by judicious treatment with soothing lotions, this may be usually subdued without much difficulty.*

If, after a week or two, the quantity of the albumen, which had been diminished by the means now recommended, again becomes increased, and

* It has been observed however, that erysipelas and other cutaneous affections are more apt to be troublesome and unyielding during the cachexia induced by nephritic disease, than in most other states of the system.

especially if there are present at the same time other signs indicative of a *recrudescence* of the renal inflammation, we should repeat the bloodletting and cupping: emollient cataplasms over the loins seem to be useful also under such circumstances.

As long as any febrile movement of the system continues, the patient should keep his bed, and drink freely of demulcent and slightly nitred beverages, for the purpose of encouraging perspiration: the use of the warm-bath also is useful. When he is permitted to leave his bed, he should be warmly clothed, with flannel next the skin, and be enjoined to avoid all exposure to currents of cold air.

After the use of bloodletting, saline purgatives are to be administered, especially if the bowels are at all constipated. If dropsy is already established, or when there is any tendency to the supervention of cerebral symptoms, we must employ more active purgatives, such as jalap, gamboge, &c.*

When the stomach and bowels are irritable, and when the patient is distressed with severe vomiting or diarrhœa, the best remedies are warm baths, small doses of opium, and the application of leeches to the anus.

The food should be light and altogether unirritating; M. *Rayer* thinks highly of milk, *pour tout nourriture,* at least for several days.

So much for the treatment of *acute* albuminous nephritis; it is simple and obvious compared with that of the *chronic* form of the disease.

In the majority of cases, all that we can hope to effect is to arrest or suspend the morbid action; the complete cure is scarcely within our reach.

Whenever we have reason to suspect the existence of active hyperæmia in the affected organ, from a feverish state of the system or from the existence of local uneasiness, the application of the cupping-glasses should be resorted to; but we must be on guard not to employ depletory measures too far, when the renal lesion is suspected to be very serious.

Great mischief may be done by injudiciously lowering the powers of the system under such circumstances, and no benefit to the disease can be rationally expected,

During the course of chronic albuminous nephritis, there is apt to be, from accidental causes, an occasional aggravation or *recrudescence* of more acute symptoms; just as we observe during the progress of pulmonary phthisis, and other organic diseases. Then again the supervention of inflammatory attacks in other organs, as of the pleura or lungs, may require blood to be drawn; but let it be remembered that, whenever such attacks are grafted, so to speak, on renal disease, much more caution in the use of venesection is necessary than under ordinary circumstances.

A most useful means to arrest the progress of chronic lesion of the kidneys is the establishment of an issue or seton in the lumbar region.

M. *Rayer* has in several cases seen decided benefit derived from the internal use of the tincture of cantharides—10 or 12 drops in an emulsion: the dropsical symptoms have disappeared at the same time under its

---

* The *pulvis jalapæ compositus* of our pharmacopœias is one of the best forms that we can use: *elaterium* also is one of the most efficacious of all purgatives under such circumstances.—*Rev.*

use.* He confesses however that it is "an uncertain remedy, and one that may prove dangerous in the hands of the inexperienced." Balsamic and terebinthinate medicines he has tried, but without good effects. Equally unsuccessful in his practice have been frictions with mercurial and ioduretted ointment over the loins.

The dropsical effusion may generally be got rid of for a time by the use of hydragogue cathartics: when the patient is much enfeebled, the addition of a ferruginous preparation to them is made with advantage. When there is gastric or intestinal irritation present, drastic medicines must be used with great caution, or be altogether omitted.

Dr. *Bright* has but little confidence in the greater number of diuretic remedies, and M. *Rayer* is inclined to agree with him; he thinks that Dr. *Christison* has greatly exaggerated their value. Squill and digitalis, while they are most uncertain in their effects upon the kidneys, often disorder the stomach and distress the patient with sickness.

M. *Rayer* speaks well of a decoction of the wild horseradish: "of all diuretics it is the one which has appeared to me to offer the best chances of success."

Vapour baths have been found useful in the chronic, as well as in the acute, form of albuminous nephritis. When the dropsical effusion is considerable, they should be administered to the patient in bed. The use of certain diaphoretic medicines, especially the *Dover* and *James's* powders, has been much recommended by English writers; but M. *Rayer* says that, after a fair trial of them, he has found them very ineffectual, and that the former often causes great sickness.

When the skin is not only dry but chilly also, the tincture of guaiacum has been occasionally found to be serviceable. In cases in which the anasarca goes on increasing, in spite of all internal remedies, it has been recommended to discharge the serosity by puncturing or scarifying the skin. The distressing, and sometimes fatal, consequences which M. *Rayer* has seen to follow these operations have led him of late to disapprove of the practice in almost every case. He admits that in dropsical effusion arising from disease of the heart it may be often employed, not only without danger, but with great benefit: not so, however, he thinks, in dropsy from disease of the kidneys, in consequence of the much greater tendency in the latter case of the punctures becoming inflamed and even gangrenous.

With respect to the treatment of those *secondary affections*, which so frequently complicate chronic albuminous nephritis, it is unnecessary to say much at present. Suffice it to state that, if they are of an acute character, they must be promptly subdued within the first twenty-four hours after their development; and, on the other hand, if they be passive or chronic in their nature, we must not expect to do more than merely to

* Dr. *Wells* treated five cases of dropsy, in which the urine was coagulable, with large doses of the tincture of cantharides—from 30 to 60 drops in the course of the twenty-four hours. In three of the cases there was a marked amendment; in one, the albumen disappeared from the urine. In the other two cases, there was no improvement. Dr. *Blackall* has remarked that the tincture seemed sometimes to increase the coagulability of the urine. It appears, therefore, that there are still some doubts as to its curative action.

palliate the more distressing symptoms. The following general directions are laid down by M. *Rayer*:—

1. Calomel in purgative doses, cupping over the mastoid region, and blisters to the nape of the neck, are the principal remedies against cerebral affections.

2. Inflammation of the bronchi, pleura, lungs, or peritoneum requires the usual antiphlogistic treatment; but the physician should remember that the weakness of the constitution, the alteration of the blood, the co-existence of dropsy, and the lesions of the kidneys and perhaps of other organs also, greatly diminish the chances of success.

3. Bitters, tonics, and creosote, are better remedies to allay the vomiting, so often caused by derangements of the urinary secretion, than ice, opium, gazeous draughts, cupping, &c.

4. Opiate preparations are most effectual against obstinate diarrhœa.

As to general regimen and diet, he lays much stress on the selection of a warm and dry locality for residence, on warm flannel clothing, on the use of light and easily digested articles of food, on the occasional use of warm aromatic baths, and on a prolonged course of steel medicines: a small portion of generous white wine may be allowed to the patient at the same time, if no symptom contra-indicates its continuance.

Having concluded this part of his subject, M. *Rayer* proceeds to describe more minutely the relations between albuminous nephritis and other diseases of the urinary organs, and also between it and various morbid lesions of other viscera and other parts of the system; illustrating his observations with the reports of numerous cases, drawn chiefly from his own experience. The cases we cannot find room for; but we shall now endeavour to lay before our readers the sum and substance of his valuable researches on this head.

"There are," says he, "several striking analogies between simple nephritis and albuminous nephritis. Both are alike produced by the impression of cold and moisture. In the acute stage, (with the exception of pus, which is exceedingly rarely, if ever, met with in the albuminous disease) every thing is common: the injection of the parenchyma of the kidneys, the increase of their bulk, the yellow discolouration of their substance, &c. In the chronic stage, when this is far advanced, the lesions are so similar that, without various circumstances drawn from the course of the diseases, from the presence or absence of dropsical effusion and of the albumen in the urine, it would be impossible to distinguish the one from the other. But, on the other hand, some strong points of dissimilarity separate the two morbid states; and one of the most striking of these is without doubt the marked influence which diseases of the urethra, bladder, prostate gland, ureters, and pelvis of the kidney have on the development of simple nephritis, while they seem to exert little or none on that of the albuminous kind. Now this circumstance points out a great difference in the nature of these two renal affections."

In simple nephritis, the pain in the loins is usually greater, and there is generally more or less of an uneasy numbness extending down the inside of the thigh; the testicle is often retracted, and micturition is more frequent and painful.

M. *Rayer* comments on several cases reported by different writers, wherein he thinks that an error of diagnosis was committed. Thus a case related by Dr. *Gregory* of albuminous nephritis coming on after the operation of lithotomy, he considers to have been one of simple nephritis, even although albumen and blood globules were found in the urine. Several of the cases too recorded in Dr. *Christison's* work, where the disease of the kidneys was associated with morbid states of the bladder, are in his opinion erroneously described as instances of the genuine *morbus Brightii*.

Alluding to one case in particular, No. XVIII. M. *Rayer* says:—" In this, as in several others, Dr. *Christison* appears to me to infer too readily the existence of the granular affection of the kidneys from the mere presence of albumen in the urine, although he is not ignorant that this phenomenon is common to several other maladies of the urinary passages."

A morbid state of the pelvis and calices of the kidneys is of much more frequent occurrence in simple than in albuminous nephritis; and for the reason that, in the latter disease, the inflammation is usually seated almost exclusively in the cortical substance of the affected organ. Now whenever the pelvis and calices are inflamed, there is always a morbid secretion of mucus, which mingling with the urine renders it more or less cloudy. By examining the deposit or cloudiness with the microscope, we may recognise the mucous globules and the debris of epithelium. Simple nephritis is often the result of a previous disease of the pelvis and calices; the albuminous kind is rarely or never.

The occurrence of particles (grains) of purulent matter in kidneys affected with genuine *morbus Brightii* is, according to the experience of M. *Rayer*, very rare; and, when he has met with them, he has always considered the phenomenon as the result of an accidental inflammatory attack of the organ.

### *Relations of Albuminous Nephritis with Diseases of the Heart and Pericardium.**

Our author is of opinion that the influence of renal disease on the development of these maladies has been much exaggerated by English writers. He has met with numerous cases in which there was a co-existence of the two morbid affections, although not in the proportion observed by *Bright*;† but, in not a few of them, the cardiac disease was evidently antecedent to the disease of the kidneys. " If these were deducted from the entire number, there would remain a very small number indeed which could be

---

* One of the sections in this chapter in *Rayer's* work is headed " Albuminous Nephritis simulated by Diseases of the Heart."

The kidneys, as well indeed as all the other viscera, are liable to become congested with blood whenever there is any embarassment of the circulation; when this is the case, the urine is apt to be charged with albumen, but its specific gravity is not much, if at all, affected, and the urea and the urates are not decidedly diminished.

† Dr. *Bright* says, that in 65 out of 100 cases of granular affection of the kidneys, was the heart found to be more or less diseased, and that in about one-half of these (65) cases it was remarkably so. M. *Rayer*, on other hand, says, that in not more than about a fifth of the cases which have come under his notice, he found, during life or after death, any notable alterations in the heart or pericardium.

considered as affections genuinely *secondary*, or owing their origin to a lesion of the kidneys;" and he adds, "Were I to judge from the results of my own observations, I should say that albuminous nephritis is most frequently added to cardiac affections, and is a secondary affection of them."

That the urine is often albuminous in patients affected with disease of the heart, when there is no serious affection of the kidneys, cannot be disputed by any experienced physician; but if, along with having this character, it is of a pale citrine colour, and of a low specific gravity, we have strong presumption that there is genuine renal degeneration existent.

With respect to the lesions of the pericardium that are unusually associated with the morbus Brightii, M. *Rayer* commences his remarks by stating that, "often in the course of this affection the pericardium contains a notable quantity—four or five ounces—of limpid serosity; but it is rare that the amount of this is so considerable as to constitute a genuine hydro-pericardium."

Every pathologist knows that, whenever there is a disease of the heart, there is always a strong disposition to its investing membrane becoming affected with inflammation or with dropsical effusion. We must therefore be upon our guard not to attribute these lesions on all occasions to the existence of renal disease, when there happens to be present at the same time any affection of the heart.

It is often difficult, as we have already stated, to determine as to the relative antiquity of cardiac and renal disease, when co-existent, and to decide which organ was primarily affected. Of this, however, we may be quite assured that, in all cases where the kidneys are diseased, the disposition to inflammation of the pericardium, and other organs, is greatly increased.

Hence, the importance of the physician having his attention frequently directed to examine the state of the heart, whenever the urine is found to be in an abnormal condition.

The remarks, which we have now made as to pericarditis in patients affected with renal disease, are equally applicable to the inflammation of the lining membrane of the cavities of the heart, or endocarditis.

Before closing our remarks on the connexion of renal disease with the organs of circulation, we may state that, in several cases, the bloodvessels of the affected kidneys have been found to exhibit certain lesions which seem often to have been induced by the extension of the inflammation of the cortical substance.

Thus the renal veins are sometimes considerably thickened, and their cavity filled with fibrinous clots. M. *Rayer* confirms the observations of Dr. *Bright*, that the small divisions of the renal artery are less easily penetrated by an injection thrown into them, than in a healthy state.

### *Relations of Albuminous Nephritis with the Diseases of the Respiratory Organs.*

We are prepared, from the contents of the preceding pages, to anticipate that the lungs and their appendages will frequently be affected, when the kidneys are diseased; for when are the central organs of circulation ever disordered, but there is a marked tendency to various morbid states of the pulmonary apparatus? Whoever has read the beautiful disquisition of Mr. *Wardrop,* in his work on the Heart, on what he has called the *pulmo-cardiac function,* will remember many striking illustrations of this fact.

M. *Rayer* says;—"I have related several examples of the coincidence of pulmonary disease with simple nephritis. This complication is much more frequent in albuminous nephritis; and this frequency is such that it is impossible not to admit the influence of this disease on the development of certain lesions of the lungs and pleura, and, on the other hand, the no less remarkable influence of chronic pulmonary diseases on the affections of the kidneys."

We shall first allude to *Bronchitis.*

This is certainly the most frequent of all the complications or *secondary* affections attending chronic albuminous nephritis; it was present in seven eighth's of M. *Rayer*'s cases. The bronchitis is generally in a chronic or passive form itself; and frequently there is so little distress in the breathing that the patient takes little or no notice of it. Not a few patients, however, labouring under renal disease, are cut off by a sudden aggravation of the bronchitic symptoms. As might be expected, little can be done by medicine, except to relieve the more urgent distress: we cannot hope to get rid of the disease altogether.

*Pneumonia* also is a not unfrequent secondary affection of several renal diseases, such as simple nephritis, albuminous nephritis, and diabetes. It is very often of the lobular kind, so well described by *Laennec* and other recent writers. Its symptoms are usually more or less masked in consequence of the cachexia of the constitution; and it has been found that the stethoscopic signs have in many cases been very unsatisfactory or even fallacious. It is unnecessary therefore to say how cautious should the physician be in watching his patient. If the pneumonic attack be acute, he will probably sink under it, whatever treatment be adopted. M. *Rayer* thinks favourably of the tartar-emetic in full doses, in such cases. Besides bronchitis and pneumonia, *pleurisy,* with or without effusion, is not unfrequently developed; occasionally also *œdema of the lungs, pulmonary apoplexy,* &c.

M. *Rayer* dwells at considerable length on the connexion between *phthisis* and albuminous nephritis, and is of opinion that the latter disease is much more frequently induced by tubercular cachexy, than *Bright* and some other writers seem to suppose. Certain it is that the urine is often found to be albuminous during the course of phthisis, and that the kidneys, if not exhibiting the necroscopic appearances of genuine granular degeneration—and even these are occasionally present—are usually more or less diseased. The co-existence of any renal disorder will always aggravate the pulmonary affection, and probably hasten on the fatal termination. M. *Rayer* remarks that in several cases he has observed the colliquative sweating to cease altogether, when any œdema of the limbs took place in a phthisical patient. On the whole he should say, from the results of his own experience, that albuminous nephritis was more frequently a secondary affection of phthisis than *vice versâ.*

*Relations of Albuminous Nephritis with Diseases of the Alimentary Passages.*

There are various diseases of these passages that are very frequently developed during the course of albuminous nephritis, of which they seem to be the results and consequences. Occasionally aphthæ appear in the mouth and fauces; the English physicians, who have been too apt to have recourse to mercury in renal disease, have observed that the gums, &c. are more readily affected with this mineral in it than in other disorders.

Œdematous angina, a very fatal complaint, has been noticed in several cases, and especially in those which have followed attacks of scarlet fever.

The functions of the stomach are very commonly disturbed more or less severely; nausea and vomiting being present in a considerable proportion of cases, more especially in the latter stages of the disease. Small doses of magnesia, lime water, hydrocyanic acid, or creosote—given a few minutes before eating or drinking—will sometimes check this troublesome symptom. Opium, æther, brandy, and such like medicines, in certain cases, answer better. If the vomiting is not arrested by the use of these means, the application of a blister or of a moxa to the pit of the stomach will sometimes succeed.

In two cases M. *Rayer* found on dissection an extensive *ramollissement* of the mucous membrane of the stomach. When this lesion exists, the vomiting is usually observed to be aggravated by taking anything into the stomach, whether food or medicine. Under such circumstances enemata and the endermic exhibition of remedies must be trusted to.

Diarrhœa is a symptom which still more frequently accompanies disease of the kidneys than even vomiting; it was present in more than one half of the cases observed by M. *Rayer*. However profuse it may be, it seldom induces any diminution in the dropsical effusion. On dissection, we usually find patches, more or less extensive, of great vascular injection in different parts of the small intestines; the mucous membrane is sometimes much softened, and occasionally exhibits a deep chocolate colour. Ulcerations here and there are not unfrequently observed, and the mucous follicles are apt to be puffy and swollen: in some cases however the only morbid appearances discoverable is an anæmic pallor of the intestines. Dr. *Christison* recommends a pill composed of three grains of the acetate of lead and half a grain of opium, to be given three, four, or even six times a day.

*Peritonitis* is one of the least frequent of the secondary affections induced by albuminous nephritis.

Morbid changes of the *liver* are not so often co-existent with renal disease as might have been expected. *Bright* states that in eighteen cases only out of 100 of granular degeneration of the kidneys did he find any serious alteration in the texture of the liver; and the results of *Rayer's* researches seem to lead to a similar conclusion. In almost all the cases—viz. where the liver was decidedly diseased—the spleen was observed to be more or less altered from its normal condition.

*Relations of Albuminous Nephritis with Cerebral Affections.*

"Cerebral affections," says M. *Rayer*, "occur but rarely in the course of this disease; but often, shortly before death, there supervenes a state of coma, analogous to what is not unfrequently observed during the progress, and especially towards the close, of a number of diseases of the kidneys, and more particularly of grave simple nephritis.* Attacks of apoplexy, coma, epileptiform convulsions, and sudden death—either without any appreciable lesion of the encephalon, or, which is more frequent, with subarachnoid and ventricular effusion—are the most common complications of albuminous nephritis; but they seem to be rather of an accidental than of a genuinely secondary or consecutive character." Whenever they do occur, they must always be regarded as very alarming symptoms. It is by no means necessary that the urinary secretion be suppressed, or even at all diminished, before a state of complete coma comes on. *Christison* assures us that he has seen it to supervene and prove speedily fatal, when the patient was passing from two to three pints of urine daily; and, on the other hand, he has observed in some cases the secretion to be exceedingly reduced in quantity, without any threatening of cerebral symptoms. Coma rarely occurs in the early stage of the disease, except indeed where this has followed upon an attack of scarlet fever. We may again mention that Dr. *Barlow* obtained nitrate of urea from the fluid effused into the cerebral ventricles of a patient who, while labouring under disease of the kidney, died from an attack on the brain.

*Relations of Albuminous Nephritis with Eruptive Fevers.*

The condition of the urinary secretion during the course of the regular exanthemata has hitherto met with but little attention from medical men;—except indeed during scarlet fever. In some cases of measles and also of small-pox, as well as of scarlet fever, the urine is observed to contain a certain quantity of blood, and consequently of albumen; and, after death, the kidneys have been found gorged with dark blood, and occasionally coagula have been found in their pelves. M. *Rayer* has knowledge of one case of albuminous nephritis occurring during the period of convalescence from confluent small-pox; and Dr. *Gregory* has cited a similar case, attended with dropsical effusion, following an attack of measles. But such instances are, at least as far as we yet know, only of casual occurrence; the subject however, it must be admitted, requires much more attentive examination than it has yet received, before we can decide how far either of these eruptive fevers has a tendency to induce the development of renal disease. As a sequela of scarlatina, albuminous nephritis, both in its acute and in its chronic form, is by no means an unfrequent phenomenon.

M. *Rayer* examines the connexion between these two diseases at great length, and, we need scarcely add, with admirable candour and ability. This chapter of his work is exceedingly worth an attentive perusal, and had our limits allowed, we should have most gladly followed him through his interesting description. The two important conclusions, which he draws from his researches are these:

---

* *Rayer* alludes, in terms of praise, to a paper read by Dr. *Wilson* before the London College of Physicians in 1835, and also to a valuable communication by Dr. *Addison*, published in the Guy's Hospital Reports for April 1839.

1. That in certain cases of scarlatina—especially during its later stage, that of the desquamation of the skin,—the urine is more or less highly charged with albumen, without dropsy supervening; the kidneys, under such circumstances, being found on dissection, to be highly congested with blood, and exhibiting lesions which altogether correspond with those, already described as characteristic of the first form of albuminous nephritis, induced by exposure to cold and damp or by the abuse of spirituous liquors.

2. That the anasarca, which frequently follows scarlatina*—by its mode of development and prognosis, by its occasional cause (the impression of cold and moisture), by its general diffusion, by the alteration of the urinary secretion which accompanies it, by its abdominal, thoracic, and cerebral complications, by the lesions discoverable on dissection, and by the results of remedial treatment—cannot but be considered analogous with the dropsy supervening on acute or chronic albuminous nephritis, when this has been induced by other causes.

When albuminous nephritis is developed after an attack of scarlatina, it usually appears towards the close of the third, or the commencement of the fourth week after the appearance of the eruption. The patient, who for some days previously may have been very well, becomes uneasy and somewhat feverish; the sleep is disturbed, the appetite is impaired, and sometimes nausea and vomiting are present. A few days afterwards a puffiness is noticed about the eyelids; this gradually extends to the rest of the face and to the neck, and thence to the extremities and trunk; in some cases, the œdema shews itself almost quite suddenly over the whole surface of the body at the same time. The urine is almost always much diminished in quantity; and is usually voided frequently and with some difficulty; it is of a deep reddish-brown colour, and often contains a portion of blood mixed with it. Suspended in it may be seen a flocculent whitish matter, resembling unclarified whey, or (when there is any admixture of blood in the urine) the water in which raw flesh has been macerated. Its specific gravity will generally be found more or less below the normal standard. There is usually considerable pyrexia present; the action of the heart is strong or even tumultuous; and the breathing is quickened and oppressed. In some cases the head, in others the chest, and in a third set the abdomen is the seat of the chief suffering and danger.

The acute form of albuminous nephritis has been known to come on very suddenly after scarlet fever, when the patient has been incautiously exposed to cold, and to prove fatal within forty-eight hours after the attack, either from convulsions, or coma, or asphyxia supervening.

When the disease has the passive form from the first, there is often little or no pyrexia present; but the patient does not recover his strength or

* "I here speak," says M. *Rayer*, "of the dropsy which is most usually observed; for it is well known that, after scarlet fever, we sometimes meet with some forms of dropsical effusion induced by other causes (affections of the heart, diseases of the liver), and which cannot be attributed to any morbid state of the kidneys."

appetite: his sleep is disturbed, and there is a general uneasiness. By and bye, the face or the extremities are observed to be somewhat œdematous; the breathing perhaps becomes oppressed; and the anasarca becomes more and more diffused. If the urine be examined, it will be found in almost every case—the exceptions are exceedingly rare—to be albuminous, and to be of a lower specific gravity than in health. Whenever the urinary secretion is in this state, the physician ought to be on his guard, whether dropsical symptoms have made their appearance or not; for some cerebral* or thoracic affection may suddenly supervene and cut the patient off in a day or two, in spite of the most active remedies.

It is scarcely necessary to dwell upon the mode of treatment to be followed in albuminous nephritis, whether it be accompanied with dropsy or not, after attacks of scarlet fever; as this must be conducted on quite the same principles as those which we have explained in a previous part of this paper. Whether the disease be of the acute or of the passive and chronic form, the patient should be kept in bed, and perspiration be promoted by the use of vapour or of warm baths, and of tepid demulcent drinks. Bleeding, general as well as local, will be required according to the character of the existing symptoms. Drastic purgatives and stimulating powerful diuretics are on the whole hurtful; the milder forms of these remedies are decidedly better. When the disease is considerably protracted, and at the same time is unattended with fever, recourse should be had to the use of tonics, as steel or quinine in some bitter infusion, and of wine.† The repeated application of blisters over the loins has in many cases greatly promoted the quickness of the cure.

M. *Rayer* does not allude to the administration of mercury under any form in the treatment of renal disease, whether this be of an acute or of a passive character: we may therefore suppose that he does not approve of it.

Before quitting this part of our subject, that regards the connexion of albuminous nephritis with the pyrexiæ, it may be well to state that, in typhus and also in yellow fever, the urine has been occasionally found charged with a certain portion of albumen, with or without blood globules; but this symptom alone is not, it is to be remembered, pathognomonic of the existence of albuminous nephritis, unless the other peculiar abnormal changes in the secretion be present at the same time; and such is seldom the case. Moreover dropsy is of very rare occurrence indeed after either of these forms of fever.

---

* Not a few cases of the *wasserschlag*, or sudden hydrocephalus, have occurred under such circumstances.

† Dr. *Hamilton* records a very instructive case of dropsy after scarlatina treated with stimulants. He was called in great haste to see a young child, who was dangerously ill with dropsy after this disease; the extremities were cold, the lips livid, and the breathing exceedingly oppressed: indeed Dr. H. did not expect her to survive the night. He ordered a blister to be applied on the chest, and as much punch, made with gin or whiskey, to be given to the child as she could be made to drink. These remedies quickly induced a great amendment; the urine became much more copious, and all the unfavourable symptoms subsided: ultimately she quite recovered.

After intermittent fevers, on the contrary, dropsical effusion, into the general cellular substance and into the abdominal cavity, is by no means unfrequent. It is now well known that dropsy, occurring under such circumstances, is by no means always of the same nature or yields to the same mode of treatment, as has been hitherto too generally believed. In one case, the œdematous swelling of the feet and of the face quickly disappears under the use of quinine and steel: the disease, in such cases, seems to be generally connected with enlargement of the spleen. In another case, the dropsy, it would appear, is connected with a renal affection, the urine being found to be more or less highly albuminous, and the disease exhibiting the same symptoms and the same general progress as we observe usually in albuminous nephritis. Such cases are to be treated on the principles explained in a former part of this article; but, should there be a co-existing enlargement of the spleen, or any return of the agueish symptoms, the administration of bark will be required at the same time.

M. *Rayer* devotes a few pages to point out the not unfrequent connexion of renal disease, and an albuminous condition of the urinary secretion with scrofulous maladies. If such patients have been exposed to wet and cold, or are of intemperate habits, the disposition will be greatly increased.

The venereal cachexia also is, according to our author's experience, a powerfully predisposing influence to the development of albuminous nephritis. *Wells, Blackall,* and some other writers, have attributed the disease, under such circumstances, rather to the action of mercury than of syphilis on the system; but M. *Rayer* leans to the opposite opinion. This point requires further examination, before we can arrive at any exact conclusions.

"There is one circumstance," says our author, "that I wish to direct the reader's attention to, and it is this; in almost every case of chronic albuminous nephritis which I have met with in patients affected with constitutional syphilis, the liver was in an unhealthy condition. Had it become so from irregularities of diet, or from the abuse of spirituous drinks? This I have not been able to determine in many of the cases. I may add that, having observed pretty frequently similar maladies of the liver, but without any lesion of the kidneys, in persons affected with constitutional syphilis, I have been led to the opinion that these alterations of the liver appear to me to be connected with the syphilitic cachexia. When the liver is diseased, but without any co-existing renal affection, the urine is usually scanty, and of deep red colour, and deposits a lateritious sediment, even when ascites is present; on the contrary, when the kidneys are simultaneously involved, the urine is of pale yellow colour, more or less charged with albumen, and without any brick-dust sediment. I know few diseases, which have less chance of any relief than these complicated affections of the kidney and liver occurring in a venereal habit of body: a good deal however may sometimes be done by a judicious use of the tisan of Feltz, the pills of Sedillot, and some of the preparations of opium."

M. *Rayer* closes his account of albuminous nephritis with an elaborate historical narrative of the most striking descriptions, in the works of different writers from the time of the early Greek writers down to the period of Dr. Bright's first publication in 1827, of the diseased conditions of the kidneys most frequently found in cases of dropsy, and of the subsequent discovery of albumen being occasionally present in the urine. This narrative has been drawn up with great care and candour. It shews, in the first place, that many of the older physicians, from the time of Hippocrates himself, through the period of Arabian literature, down to the middle of last century, have repeatedly pointed out the frequent existence of disease of the kidneys in cases of general dropsy, and that it has been only within the last 60 or 70 years that the influence of such disease has been overlooked: so true is the saying, that "nothing is so new as that which is forgotten." *Hippocrates* uses these remarkable words:—"Dropsies supervening upon acute diseases are always dangerous, for they do not free the patient from fever; they increase the existing pains, and often terminate fatally. Some dropsies proceed *from the flanks and the loins*, others from the liver. In the first set the feet swell, and there is often a most obstinate diarrhœa, which however does not abate the pains in the loins, nor empty the abdominal effusion." *Ætius* is still more exact; he distinctly tells us that persons, who are affected with *induration of the kidneys*, become in the long run dropsical. *Van Helmont* repeatedly alludes to the kidney being the "artifex" and "principalis effector" of dropsy. *Bonetus*, describing the appearances found on dissection in a case of dropsy, says that "the kidneys had quite lost their natural colour, and had become of an almost milky-white colour," and *Morgagni* still more accurately describes the lesion in these words; "both kidneys were irregular in their surface, and spotted over with numerous white spots."

In 1825, M. *Andral*, in his description of a case of dropsy in which there was no existing disease of the heart or of the peritoneum, draws the attention of his readers to the state of the kidneys in the following passage: "there was, however, another organ which exhibited an alteration that must not be forgotten: this was the kidney, the cortical substance, and part also of the tubular substance of which were changed into a *whitish granular substance*, divided into small masses or grains, interposed between the remaining portions which retained their natural red hue. Had this peculiar alteration of the kidney," he asks, "caused any obstacle to the free secretion of the urine, and thereby contributed, in a manner more or less direct, to the production of the dropsy? However this may be, it was the only lesion discoverable on dissection; but if the cause of the disease in this case is obscure, the cause of death is very apparent; it was evidently owing to the double hydrothorax."

The second part of M. *Rayer's* historical narrative gives an account of the various publications, which notice the occasional presence of albumen in the urine, from the time of *Cotugno*, in 1770, down to the present time. He mentions with especial praise the memoir of *Cruickshank*, in 1798, and the papers by Dr. *Wells*, in the Transactions of a Society for the improvement of Medical and Surgical Knowledge, which he remarks are "trop peu connus" in France. The works of *Blackall, Nysten, Prout*, (l'auteur des travaux les plus remarquables faits sur les alterations de l'urine dans ces dernier temps), &c. are successively noticed. The reader is thus brought down to the period when Dr. *Bright* published "son beau travail," Reports of Medical Cases, in 1827. The subsequent works and papers by Drs. *Gregory, Christison, Craigie, Barlow, Copland, Hamilton, Seymour, Willis*, &c. in this country, and of MM. *Tissot, Monassot*,

*Desir, Martin Solon,* &c. in France, are each noticed and commented upon.

"Arrived," says our author, "at the end of my work on albuminous nephritis, it now only remains for me to call the attention of the reader to some points in the history of this disease. And first, he should especially attend to the marked differences which it presents in its acute and in its chronic form—differences which I have thought it my duty the more to insist upon, seeing that hitherto they have not been explained with sufficient clearness, and medical men have too long and too generally supposed that the *morbus Brightii* was an *organic* affection. This opinion has been the source of many serious errors entertained as to the nature and curability of the disease; it has given rise to discussions which, having no true foundation, could end in nothing satisfactory. The constitution of the urine is not the same in the two forms. All writers have given, as an important character of the urine in *Bright's* disease, a diminution of its specific gravity; now, not only is this character not true of the urine in the acute form of the disease, but the very reverse is most frequently the case. The diagnosis of the two forms is, besides, the more necessary as the antiphlogistic mode of treatment, so often efficacious in the acute form, is generally either quite useless or positively hurtful in the chronic form. Physiological pathology will necessarily attend much to the phenomena of albuminous nephritis in all enquiries as to the production of dropsy. It is certainly not easy to explain, in some cases, the mechanism in which this takes place. It has been supposed that the mere impoverishment of the blood from the loss of its albumen was the immediate cause; but then, if this is always the case, how comes it to pass that dropsical effusion sometimes occurs very rapidly, before the loss of the albumen of the blood is at all considerable? and that in other cases hæmaturia may exist for months and even years, and yet no dropsy supervene?

Lastly, I would again impress the extreme importance of using all means to guard against the influences of cold and damp in every case where the urine has once exhibited the phenomena of albuminous nephritis; many a relapse might be avoided by this precaution."

We take leave of M. *Rayer's* work with the deepest respect for the talents, the industry, and candour of its author. Besides his high merits as a clinical physician, he is a "ripe and good" scholar, well informed in the medical literature of other countries as well as of his own. We know of no foreign work that better deserves a translation, compressed and annotated as a matter of course, than this *Traité des Maladies des Reins.*

